# Analysis of Potential Mechanism of Herbal Formula Taohong Siwu Decoction against Vascular Dementia Based on Network Pharmacology and Molecular Docking

**DOI:** 10.1155/2023/1235552

**Published:** 2023-01-23

**Authors:** Ruixin He, Fengjiao He, Zhenliang Hu, Yifang He, Xinting Zeng, Yao Liu, Liang Tang, Ju Xiang, Jianming Li, Binsheng He, Qin Xiang

**Affiliations:** ^1^Department of Basic Biology, Changsha Medical University, Changsha, China; ^2^Center for Neuroscience and Behavior, Changsha Medical University, Changsha, China; ^3^Academics Working Station, Changsha Medical University, Changsha, China; ^4^Department of Rehabilitation, Xiangya Boai Rehabilitation Hospital, Changsha, China

## Abstract

Vascular dementia (VaD) is the second most prevalent dementia, which is attributable to neurovascular dysfunction. Currently, no approved pharmaceuticals are available. Taohong Siwu decoction (TSD) is a traditional Chinese medicine prescription with powerful antiapoptosis and anti-inflammatory properties. In this study, a network pharmacology approach together with molecular docking validation was used to explore the probable mechanism of action of TSD against VaD. A total of 44 active components, 202 potential targets of components, and 3,613 VaD-related targets including 161 intersecting were obtained. The potential chemical components including kaempferol, baicalein, beta-carotene, luteolin, quercetin, and beta-sitosterol involved in the inflammatory response, oxidative stress, and apoptosis might have potential therapeutic effects on the treatment of VaD. The potential core targets including AKT1, CASP3, IL1*β*, JUN, and TP53 associated with cell apoptosis and inflammatory might account for the essential therapeutic effects of TSD in VaD. The results indicated that TSD protected against VaD through multicomponent and multitarget modes. Though the detailed mechanism of action of various active ingredients needs to be further illustrated, TSD still showed a promising therapeutic agent for VaD due to its biological activity.

## 1. Introduction

Vascular dementia (VaD) is one of the most prevalent types of dementia involved in a series of symptoms that are mainly characterized by progressive neurocognitive impairment attributed to various cerebrovascular pathologies [1, 2]. VaD is responsible for approximately 15-20% of dementia cases in China, placing a tremendous burden on society and patients' families [3]. A growing amount of evidence indicated that the pathogenesis of VaD was heterogeneous and involved multiple interactions of various physiological functions of the brain [1]. Notably, an increasing number of studies discovered that the causes for VaD were a series of structural and functional dysfunction in the cerebral neurovascular unit which encompassed sealed endothelial cells forming the blood-brain barrier, perivascular cells including pericytes, vascular smooth muscle cells, astrocytes, microglia, and neurons [4, 5]. The structural damage of neurovascular units frequently results in blood-brain barrier dysfunction, neurovascular cell uncoupling, inflammatory response aggravation, and ultimately, the loss of neurons [5]. Although several drugs, including donepezil, galantamine, and memantine approved for AD, have been proved to be suitable for alleviating some major symptoms of VaD, these agents remain a huge challenge and hurdle for preventing or delaying the process of VaD [6]. Accordingly, it is essential to develop more effective therapeutic drugs and to clarify the mechanism of action of drugs in treating VaD clinically.

Traditional Chinese medicine (TCM), as a major component of alternative and complementary medical remedies, has attracted increasing attention worldwide due to its high clinical effectiveness and security [7]. It was believed by the theory of TCM that VaD was a result of a deficiency of essential Qi, along with a toxin substance damaging brain collaterals [3]. Consequently, the principle of treatment was to remove the accumulated toxigenic products, eliminate blood stasis, and promote blood circulation and nourishment. An increasing number of herbs, including *Radix Salviae* (Danshen in Chinese), *Huperzia serrata* (Qiancengta in Chinese), *Ligusticum chuanxion*g (Chuanxiong in Chinese), *Ginkgo biloba* (Yinxing in Chinese), and *Panax ginseng* (Renshen in Chinese), have been revealed the clinical pharmacological effects on dementia and dementia-like disorders [3, 6, 8].

Taohong Siwu decoction (TSD), a multiherbal formula, was initially documented in Wu Qian's “Yi Zong Jin Jian” (Qing Dynasty of China). The formula is composed of six herbs including *Paeoniae Radix Alba* (Baishao in Chinese), *Rehmanniae Radix Praeparata* (Shudihuang in Chinese), *Angelicae Sinensis Radi*x (Danggui in Chinese), *Chuanxiong Rhizoma* (Chuanxiong in Chinese), *Persicae Semen* (Taoren in Chinese), and *Carthami Flos* (Honghua in Chinese). As a Chinese medicine prescription, it has been used primarily to treat deficiency and stasis of qi and blood, which are the main causes of dementia [9]. Increasingly, evidence reported that it had multiple pharmacological activities, including inhibition of cell oxidation, apoptosis, and reducing the release of inflammatory factors [10–13]. Although the chemical constituents of TSD have been documented, the major active components and target-level signaling pathways in the treatment of VaD are poorly understood. Therefore, it is of great value to study the mechanism of action of TSD resisting VaD.

In TCM, network pharmacology is a common research approach. Based on network analysis, it could reveal potential mechanisms of multi-ingredient action of Chinese herbal compounds as a whole in treating diseases. In the process of network pharmacological analysis, herbs and their targets are discovered by the Traditional Chinese Medicine Systems Pharmacology Database and Analysis Platform (TCMSP), which has proven successful in drug discovery as one of the most prevalent system pharmacology platforms for Chinese herbal medicines [14]. Despite the complexity of herbal medicine extract compound, this approach is still convenient and time-saving to initially illustrate the target and pathway relationships between drugs and diseases and also to discover potential novel targets to guide wet lab experiments in follow-up studies. In this study, a few potential bioactive compounds against VaD in TSD were identified, and the mechanisms were preliminarily elucidated based on network pharmacology and bioinformatics, which provided a certain theoretical foundation for in-depth research and clinical practice in the future.

## 2. Materials and Methods

### 2.1. Chemical Ingredient Screening in TSD

Chemical ingredients of six herbs, including *Paeoniae Radix Alba* (Baishao in Chinese), *Rehmanniae Radix Praeparata* (Shudihuang in Chinese), *Angelicae Sinensis Radi*x (Danggui in Chinese), *Chuanxiong Rhizoma* (Chuanxiong in Chinese), *Persicae Semen* (Taoren in Chinese), and *Carthami Flos* (Honghua in Chinese) in TSD prescription (the contents of herbs = 9 g: 10 g: 8 g: 6 g: 15 g: 15 g, sequentially), were retrieved from the TCMSP database [15]. The parameters of oral bioavailability (OB value) ≥ 30% and drug-like property (DL value) ≥ 0.18 were considered as herb screening and evaluation conditions for the ingredients based on recommendations from the TCMSP database and previous research [14, 16]. Subsequently, the name and molecular structure of ingredients were verified in the PubChem database (https://pubchem.ncbi.nlm.nih.gov/).

### 2.2. Target Analysis of Ingredients

The potential targets of bioactive ingredients in six herbal medicines were retrieved from the SwissTargetPrediction [17]. The SMILE structural formula form for each ingredient was uploaded, and the organism of “*Homo sapiens*” was chosen for the prediction results. The indexes with high probability (above 0.65 for 2D or above 0.85 for 3D) were selected as potential targets of the ingredients within TSD according to the SwissTargetPrediction interpretation. Eventually, the UniProt Knowledgebase (https://www.uniprot.org/) was employed to convert protein names into corresponding gene symbols.

### 2.3. Identifying Known Targets for VaD

The VaD-related gene targets were screened from four databases: GeneCards: the human gene database, DrugBank Online database, Online Mendelian Inheritance in Man (OMIM) database, and PharmGKB database with the keywords of “vascular dementia” and “*Homo sapiens*.” All disease targets were merged, duplicate items were excluded, and then VaD-related target genes were reserved for further research.

### 2.4. Target Intersection between Compounds and VaD

The Venn R package was used to compile a Venn diagram following the acquisition of the TSD and VaD targets. The intersection targets were considered as a potential target gene set for TSD to exert therapeutic effects on VaD.

### 2.5. Enrichment Analyses of Gene Ontology (GO) and Kyoto Encyclopedia of Genes and Genomes (KEGG)

Enrichment analyses of GO and KEGG were conducted through the Database for Annotation, Visualization and Integrated Discovery (DAVID) v 6.8 (https://david.ncifcrf.gov/) to annotate the function of target genes and disclose potential biological pathways of the active constituents of TSD. Terms with a threshold value of *P* ≤ 0.05 were conducted for further research, and then, the number of enriched genes was ranked within descending order. The clusterProfiler R package was conducted to demonstrate advanced bubble diagrams. Through enrichment analysis, the potential biological processes and pathways via which TSD had a therapeutic effect on VaD could be predicted.

### 2.6. Networking of Compound-Target and Pathway-Target

To provide insight into the mechanisms of action of TSD in treating VaD, networks of compound-target and pathway-target were conducted. In these networks, nodes symbolize constituents, targets, or pathways, while edges refer to links between them. To visualize networks, the Cytoscape 3.7.1 software (https://cytoscape.org/) was utilized in this research.

### 2.7. Networking of Protein-Protein Interactions (PPI) and Screening of Core Genes

The intersection targets of TSD and VaD were entered into the STRING database (https://cn.string-db.org/) to construct the PPI network. Target proteins restricted to the species of “*Homo sapiens*” and isolated proteins in the PPI network were concealed. For the purpose of understanding the relationship between proteins, the network topology was generated using the Cytoscape 3.7.1 software. The nodes in the network refer to the targets, while the edges refer to the PPI. A circle was formed based on these parameters for each protein. High centrality values and degrees implied a major role in the network. The probable core targets were then selected using the plugins MCODE and CytoHubba [18].

### 2.8. Molecular Docking for Evaluating Component-Target Interactions

Molecular docking was conducted to predict the binding affinity between active ingredients and their hub targets through the AutoDock Vina software. Structures of candidate active ingredients and targets were obtained from TCMSP and PDB databases, respectively. Dehydration, hydrogenation, and ligand separation were accomplished using the PyMOL software (https://pymol.org/2/). Docking was performed via AutoDock Vina software according to the manufacturer's process. The molecule with energy-minimized conformation in the docking was selected, and the interaction between ingredients and targets was visualized via the PyMOL software [19].

## 3. Results

### 3.1. Analysis and Prediction of TSD Targets for Treating VaD

**Figure 1:**
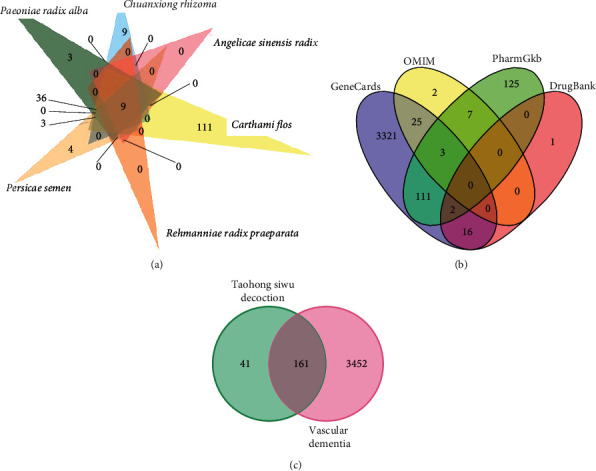
Identification of cotargets in Taohong Siwu decoction and vascular dementia. (a) Venn diagram of herbs from Taohong Siwu decoction target genes. (b) Venn diagram of vascular dementia target genes from the GeneCards, OMIM, PharmGKB, and DrugBank databases. (c) Venn diagram of cotargets in Taohong Siwu decoction and vascular dementia.

All compounds of Chinese herbs in TSD were screened based on the conditions set in Methods in the TCMSP database. Lastly, 13 compounds in *Paeoniae Radix Alba*, 7 compounds in *Chuanxiong Rhizoma*, 2 compounds in *Angelicae Sinensis Radi*x, 22 compounds in *Carthami Flos*, 2 compounds in *Rehmanniae Radix Praeparata*, and 23 compounds in *Persicae Semen* were identified, respectively. There were a total of 44 compounds screened in 6 kinds of Chinese herbs in TSD (Table 1). According to the target screening of the compounds, 79 targets in *Paeoniae Radix Alba*, 29 targets in *Chuanxiong Rhizoma*, 45 targets in *Angelicae Sinensis Radi*x, 189 targets in *Carthami Flos*, 28 targets in *Rehmanniae Radix Praeparata*, and 43 targets in *Persicae Semen* were identified, respectively. Eventually, there were a total of 202 target genes obtained in 6 Chinese herbs in TSD (Figure 1(a)). Genes related to VaD were successfully disclosed from the GeneCards, OMIM, PharmGKB, and DrugBank databases. 3,613 target genes were retrieved (Figure 1(b)). Incorporating potential targets of chemically active components in TSD prescription with those of VaD revealed exactly 161 therapeutic targets (Figure 1(c)).

### 3.2. Enrichment Analyses of GO and KEGG

**Figure 2:**
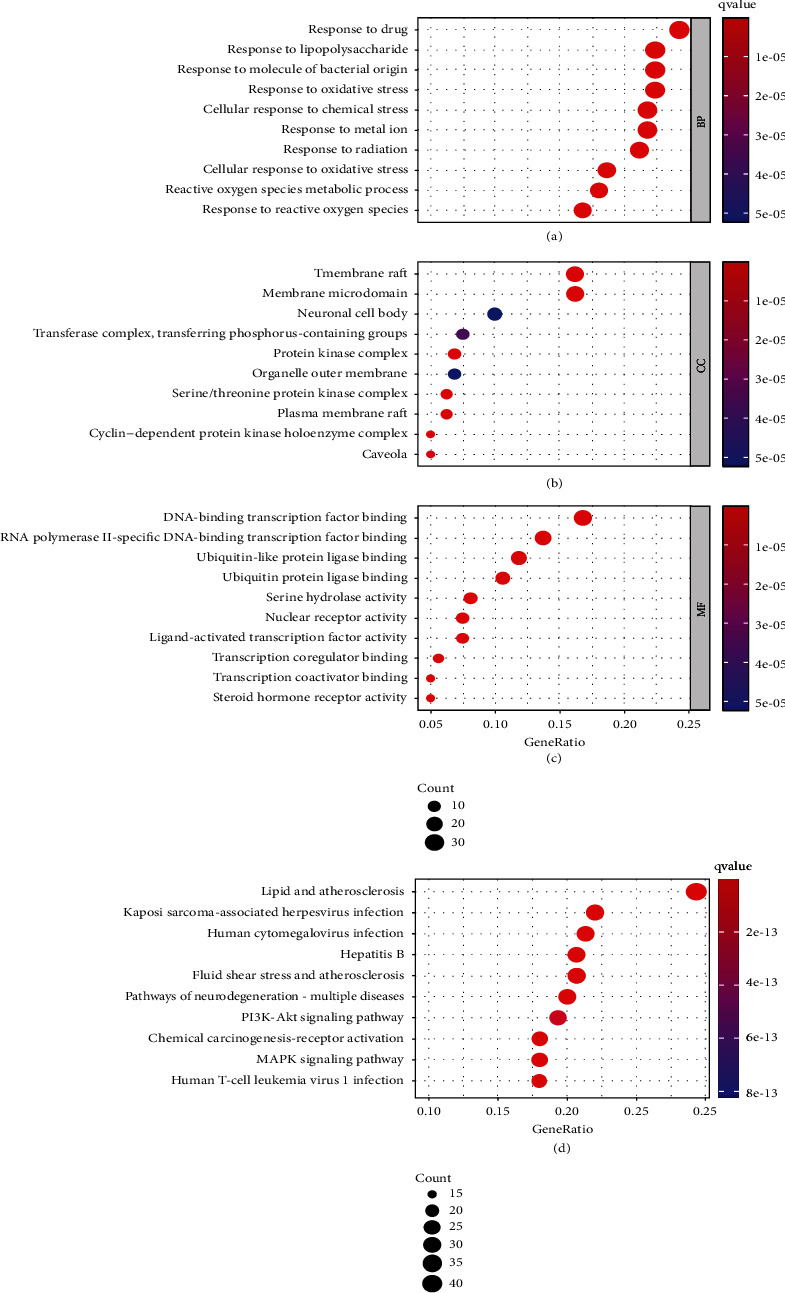
GO enrichment and KEGG enrichment analyses of intersection targets from the Taohong Siwu decoction for vascular dementia treatments. (a–c) Bubble diagram of GO function enrichment of intersection targets. (d) Bubble diagram of KEGG enrichment of intersection targets. The more enriched the targets, the larger the dots; the higher the *P* value, the bluer the dots.

According to GO enrichment analysis of the DAVID database, a total of 2,504 GO items including 2,227 biological process (BP) items, 77 cell composition (CC) items, and 200 molecular function (MF) items were revealed (*P* value < 0.01) in this study. For visualization, we further selected the top 10 catalogs in each of the three categories: BP, CC, and MF (Figures 2(a)–2(c)). The top 10 catalogs enriched were visualized with a bubble chart in which the abscissa represented gene enrichment degree, bubble size indicated gene enrichment amount, and color depth indicated *P* value. According to results of BP, targets of components of TSD for treating VaD are primarily involved in response to drug, response to lipopolysaccharide, response to molecule of bacterial origin, response to oxidative stress, cellular response to chemical stress, response to metal ion, response to radiation, reactive oxygen species metabolic process, cellular response to oxidative stress, and response to reactive oxygen species. The MF items were principally related to DNA-binding transcription factor binding, RNA polymerase II-specific DNA-binding transcription factor binding, ubiquitin-like protein ligase binding, ubiquitin protein ligase binding, serine hydrolase activity, nuclear receptor activity, ligand-activated transcription factor activity, transcription coregulator binding, transcription coactivator binding, and steroid hormone receptor activity. Results of the KEGG pathway enrichment analysis revealed that targets of components of TSD were mainly involved in 180 signaling pathways related to VaD (*P* value < 0.05). The main pathways of enrichment included lipid and atherosclerosis, Kaposi's sarcoma-associated herpesvirus infection, human cytomegalovirus infection, hepatitis B, fluid shear stress and atherosclerosis, pathways of neurodegeneration-multiple diseases, PI3K/Akt signaling pathway, MAPK signaling pathway, chemical carcinogenesis-receptor activation, and human T-cell leukemia virus 1 infection (Figure 2(d)).

### 3.3. Network Analyses of Ingredient-Target and Ingredient-Pathway

**Figure 3:**
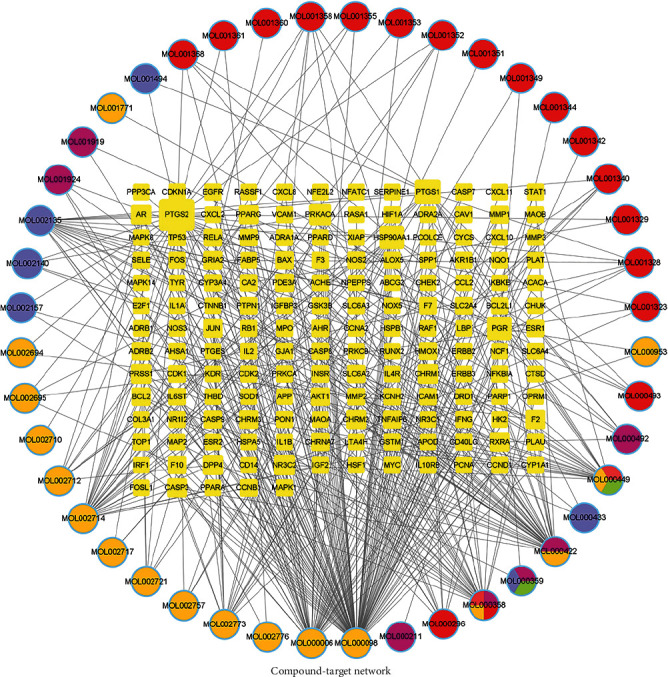
“Component-target” network. The circle nodes indicate active ingredients, and the square nodes indicate potential drug targets. The edges indicate the interactions.

**Figure 4:**
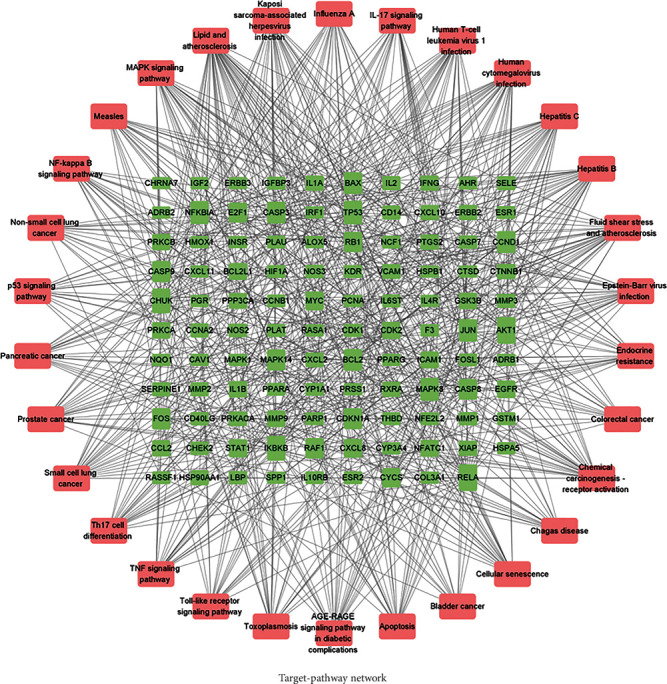
“Target-pathway” network. The red nodes indicate pathways, and the green nodes indicate potential drug targets. The edges indicate the interactions.

For building the ingredient-target network, a total of 44 active ingredients and 161 common targets were imported into the Cytoscape 3.7.1 software. As portrayed in the figure, there were 205 nodes including 44 active components and 161 targets and 428 edges in the network (Figure 3). MOL000098 (quercetin) exhibited the most target interactions (degree = 112), followed by MOL000006 (luteolin, degree = 44), MOL000422 (kaempferol, degree = 42), MOL002714 (baicalein, degree = 28), and MOL000358 (beta-sitosterol, degree = 25). These compounds with high degree nodes were speculated to play crucial roles in the pharmacologic effect of TSD. To further discover the mechanism of action of TSD alleviating VaD, a target-pathway network was reconstructed based on the top 30 significant signaling pathways and their corresponding genes. This network was comprised of 139 nodes (109 genes and 30 pathways). The NF-kappa B signaling pathway was probably the most critical of these pathways due to having the highest degree (degree = 57). Target genes, including RELA, AKT1, IKBKB, MAPK1, CHUK, NFKBIA, and TP53, were recognized as relatively high-degree targets (degree ≥ 20) (Figure 4).

### 3.4. PPI Network Analysis and Core Target Identification

**Figure 5:**
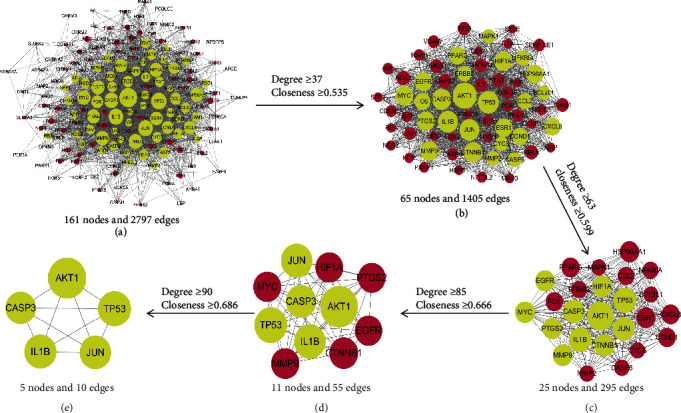
Protein-protein interaction network of intersection targets from the Taohong Siwu decoction for vascular dementia treatment. (a) All target protein interaction network diagram. The network contains 161 protein nodes and 2,797 edges. (b–e) The process for screening hub genes and topological networks. Yellow nodes have higher degrees, and a node size is proportional to a degree.

Topological analysis was employed on the PPI network to select hub nodes as key targets based on degree and closeness parameters. The STRING tool was used to illustrate the interaction between 161 targets related to VaD. As portrayed in the figure, there were 161 nodes and 2,797 edges (Figure 5(a)). The initial threshold values were degree ≥ 37 and closeness ≥ 0.535, resulting in 65 hubs and 1,405 edges (Figure 5(b)). Following the initial screen, a second screen with degree ≥ 63 and closeness ≥ 0.599, 25 hubs, and 295 edges from the 65 hubs nodes was produced (Figure 5(c)). In the third screening, the threshold values for degree and closeness were not less than 85 and 0.666, respectively. This resulted in 11 hubs and 55 edges (Figure 5(d)). Further screening with the fourth round threshold values as degree ≥ 90 and closeness ≥ 0.86 resulted in 5 hubs and 10 edges (Figure 5(e)). Finally, according to degree and closeness centrality values, AKT1, CASP3, IL1*β*, JUN, and TP53 were responsible for the top 5 core genes. These results suggested that the five high-degree hub targets might account for the essential therapeutic effects of TSD in VaD.

### 3.5. Docking Analysis of Component-Target

**Figure 6:**
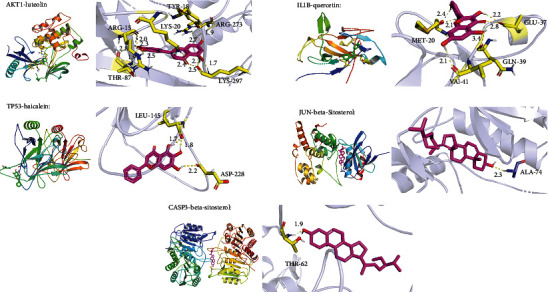
Molecular docking simulations of compounds and their core targets.

In the previous report, it was proved that two molecules exhibited a standard binding ability at binding energy less than -4.25 kcal/mol, an excellent binding ability at less than -5.0 kcal/mol, while less than -7.0 kcal/mol denoted a strong binding ability [20]. In this study, we docked five top targets (AKT1, CASP3, IL1*β*, JUN, and TP53) with six active monomers (kaempferol, baicalein, beta-carotene, luteolin, quercetin, and beta-sitosterol) of TSD. The results illustrated that most of the binding energies between two molecules were less than -7 kcal/mol, and the binding energy between beta-carotene and AKT1 was less than -12 kcal/mol (lowest) (Table 2). Molecular docking results revealed that luteolin formed hydrogen bonds with ARG-15, TYR-18, LYS-20, THR-87, ARG-273, and LYS-297 residues in AKT1 (7NH5), respectively. Baicalein formed hydrogen bonds with LEU-145 and ASP-228 residues in TP53 (5O1G), respectively. In CASP3 (3DEF), *β*-sitosterol formed hydrogen bonds with the THR-62 residue. In IL1B (5R86), quercetin formed hydrogen bonds with MET-20, GLU-37, GLN-39, and VAL-41 residues, respectively. In JUN (4Y46), beta-sitosterol formed hydrogen bonds with the ALA-74 residue (Figure 6). The remaining docking results also showed an excellent binding between drug targets and natural compounds (data not show).

## 4. Discussion

VaD arises after multiple risk factors due to primary injury in microhemodynamics and alteration in blood vessel thickening and dysfunction. As a consequence, a comparatively effective intervention for VaD should improve the cognitive function as well as reduce the production of vascular risk factors. Up to now, various herbs, including *Radix Salviae* (Danshen in Chinese), *Huperzia serrate* (Qianchenta in Chinese), *Chuanxiong Rhizoma* (Chuanxiong in Chinese), *Ginkgo Folium* (Yinxingye in Chinese), and *Panax ginseng* (Renshen in Chinese), have been widely applied in clinical practice in the treatment of VaD [3]. In the present study, we resolved around a traditional Chinese herbal medicine formula TSD derived from Siwu decoction to investigate its potential mechanism of action in treating VaD. The results demonstrated that the TSD was composed of 6 Chinese herbal medicines, contained 44 compounds, and targeted 161 genes related to VaD.

In this study, it was revealed that kaempferol, baicalein, beta-carotene, luteolin, quercetin, and beta-sitosterol in TSD might have potential therapeutic effects on the treatment of VaD based on network analysis. Kaempferol, mainly derived from *Carthami Flos* and *Paeoniae Radix Alba*, plays an important role in improving the damage of striatal neurons [21]. *β*-Sitosterol derived from *Persicae Semen*, *Paeoniae Radix Alba*, *Carthami Flos*, and *Angelicae Sinensis Radi*x is regarded to have a crucial role in potentially reducing neuron demyelination and blood lipids [21]. Luteolin, one of C*arthami Flos*' major ingredients, is involved in the inflammatory response of VaD by inhibiting the expression of AP-1 and NF-*κ*B [22]. Quercetin, baicalein, and beta-carotene are also major ingredients of *Carthami Flos* and play an important role in antioxidation and anti-inflammation, which is closely related to relevant therapeutic strategies in VaD [23]. These findings indicated that the aforementioned compounds might contribute to the foundations of the mechanism of action of TSD in treating VaD, while further research would need to be determined.

TCM therapeutics has the advantage of integrity and simultaneous capture of multiple targets and multiple pathways, which produces a stronger robust synergistic effect than any single compound. In this study, it was revealed that the therapeutic effects of TSD on VaD are mostly attributed to the potential core targets of AKT1, CASP3, IL1*β*, JUN, and TP53, which are associated with cell apoptosis and inflammatory. Through Akt activation, the p-Akt protein was produced, which inhibited apoptosis and promoted hippocampal nerve cell survival [24]. The increased levels of cleaved caspase-3 protein and activation of the caspase-3 pathway promoted the apoptosis that occurred in the progression of chronic cerebral hypoperfusion to VaD [25]. Proinflammatory mediator IL-1*β* is increasingly involved in the process of neuroinflammation, indicating that it may be strongly implicated in the progression of VaD [26]. TSD had a property of analgesia on postherpetic neuralgia rats by inhibiting TNF-*α* and IL-1*β* release, further suggesting that IL-1*β* was a potential target of TSD [12]. It was found that inhibitors of protein kinases/c-Jun amino-terminal kinase signaling pathway activated by stress significantly attenuated the Zn^2+^-induced neurotoxicity and neuronal cell death [27]. TP53 has been intensively concerned with the pathogenesis of Alzheimer's disease through mediating A*β*_1–42_ induced neurotoxicity [28]. Intriguingly, deposits of A*β* in cerebral amyloid angiopathy or cerebral blood vessels were related to vascular cognitive impairment, indicating a novel role of TP53 in VaD [1, 28]. In addition, the interactions among these core targets are also very crucial in drug interaction. Therefore, our results indicated that these five genes might be the core targets of TSD affecting VaD.

In this study, it's also revealed that the therapeutic effects of TSD on VaD are mostly related to PI3K/Akt and MAPK signaling pathways, which were closely associated with cell apoptosis and inflammation. PI3K is able to be induced by neurotrophic factors (nerve growth factor, brain-derived neurotrophic factor, glial cell line-derived neurotrophic factor, and neurotransmitters, for instance), and the phosphorylation of Akt is also enhanced after ischemia [29]. There is no doubt that the PI3K/Akt signaling pathway widely participates in central nervous system development and appears to be particularly important for maintaining neuronal cell survival, which participates in promoting B-cell lymphoma-2 (Bcl-2) expression, antagonizing apoptosis, promoting cell survival, and attenuating cell injury after ischemia and hypoxia [30]. In oxygen-glucose deprivation/reperfusion-induced PC12 cell injury, an *in vitro* cerebral ischemia-reperfusion injury model, TSD's protective effect, was significantly attenuated by inhibiting PI3K activity, which indicated that this pathway was critical to TSD protection [13]. MAPK signaling pathway was mainly involved in caspase-3-dependent apoptosis, survival, inflammation (cytokine synthesis), and stress response pathophysiological processes, which were related to neurodegenerative diseases [31]. Therefore, our results demonstrated that PI3K/Akt and MAPK signaling pathways were likely to the mechanisms of TSD affecting VaD.

TCM has been extensively used and accumulated a wealth of experience in the treatment of dementia due to its reducing adverse effects, prolonging survival time, and improving quality of life in patients. Modern studies have also revealed that a growing number of Chinese medicines have been reported to have neuroprotective effects against VaD [32]. For instance, the Bushenhuoxue formula could attenuate the level of autophagy and thereby ameliorated the learning and memory ability of VaD, while Yizhi Tongmai decoction exerted an anti-VaD role through inhibition of NLRP3 inflammasome activation [33, 34]. In a previous study, TSD was revealed to destroy the proapoptotic protein Bax expression, promote the antiapoptotic protein Bcl2 expression, reduce the lactate dehydrogenase exudation, increase the antioxidant enzyme superoxide dismutase activity, and decrease the lipid oxide malondialdehyde content in ischemic Sprague-Dawley rat model through resisting cell oxidation and apoptosis, indicating a potential protective effect on VaD [12]. In our study, network pharmacology analysis indicated that these major components of TSD were closely related to antiapoptosis and anti-inflammation as possible mechanisms in the treatment of VaD.

Network pharmacology along with bioinformatics analyses revealed the compositions and targets of TSD, as well as VaD-related genes and targets. In the study, we predicted probable therapeutic targets of TSD on VaD, revealed its action mechanisms through core compounds and genes, and provided scientific evidence for TSD to VaD. However, it is worth noting that the results from network pharmacology together with bioinformatics are a certain false-positive rate as a result of relying only on existing databases and results for network modeling. Therefore, the absence of experimental validation is the major limitation of this study. According to the findings from this study, the validation of molecular levels is of great significance to conduct in future studies.

## 5. Conclusion

In brief, to investigate the potential action mechanisms of TSD for treating VaD, compound and target prediction and map analysis were performed by using network pharmacology together with bioinformatics methods in this study. A total of 44 compounds and 202 target genes were identified in TSD and finally found 161 intersecting targets. TSD intervened VaD mainly by six active monomers (kaempferol, baicalein, beta-carotene, luteolin, quercetin, and beta-sitosterol) and five key target genes (AKT1, CASP3, IL1*β*, JUN, and TP53), which might be through two main VaD-related signaling pathways (PI3K/Akt and MAPK signaling pathways) in this network study. This study revealed potential mechanisms of TSD with multiple components, multiple targets, and multiple pathways for treating VaD, providing a scientific foundation for studying the mechanism *in vivo* and *in vitro* by wet experiment in the future.

## Figures and Tables

**Table 1:** Identification of candidate compounds for herbs in Taohong Siwu decoction.

Mol ID	Herb (Latin name)	Molecule name	Oral bioavailability (%)	Drug-likeness
MOL000422	*Carthami Flos*, *Paeoniae Radix Alba*	Kaempferol	41.88	0.24
MOL000449	*Carthami Flos*, *Rehmanniae Radix Praeparata*, *Angelicae Sinensis Radix*	Stigmasterol	43.83	0.76
MOL000359	*Chuanxiong Rhizoma*, *Rehmanniae Radix Praeparata*, *Paeoniae Radix Alba*	Sitosterol	36.91	0.75
MOL000358	*Persicae Semen*, *Carthami Flos*, *Paeoniae Radix Alba, Angelicae Sinensis Radix*	Beta-sitosterol	36.91	0.75
MOL001323	*Persicae Semen*	Sitosterol alpha1	43.28	0.78
MOL001328	*Persicae Semen*	2,3-Didehydro GA70	63.29	0.50
MOL001329	*Persicae Semen*	2,3-Didehydro GA77	88.08	0.53
MOL001340	*Persicae Semen*	GA120	84.85	0.45
MOL001342	*Persicae Semen*	GA121-isolactone	72.70	0.54
MOL001344	*Persicae Semen*	GA122-isolactone	88.11	0.54
MOL001349	*Persicae Semen*	4a-Formyl-7alpha-hydroxy-1-methyl-8-methylidene-4aalpha,4bbeta-gibbane-1alpha,10beta-dicarboxylic acid	88.60	0.46
MOL001351	*Persicae Semen*	Gibberellin A44	101.61	0.54
MOL001352	*Persicae Semen*	GA54	64.21	0.53
MOL001353	*Persicae Semen*	GA60	93.17	0.53
MOL001355	*Persicae Semen*	GA63	65.54	0.54
MOL001358	*Persicae Semen*	Gibberellin 7	73.80	0.50
MOL001360	*Persicae Semen*	GA77	87.89	0.53
MOL001361	*Persicae Semen*	GA87	68.85	0.57
MOL001368	*Persicae Semen*	3-O-p-Coumaroylquinic acid	37.63	0.29
MOL000493	*Persicae Semen*	Campesterol	37.58	0.71
MOL000296	*Persicae Semen*	Hederagenin	36.91	0.75
MOL002694	*Carthami Flos*	4-[(E)-4-(3,5-Dimethoxy-4-oxo-1-cyclohexa-2,5-dienylidene)but-2-enylidene]-2,6-dimethoxycyclohexa-2,5-dien-1-one	48.47	0.36
MOL002695	*Carthami Flos*	Lignan	43.62	0.65
MOL002710	*Carthami Flos*	Pyrethrin II	48.36	0.35
MOL002712	*Carthami Flos*	6-Hydroxykaempferol	62.13	0.27
MOL002714	*Carthami Flos*	Baicalein	33.52	0.21
MOL002717	*Carthami Flos*	qt_carthamone	51.03	0.20
MOL002721	*Carthami Flos*	Quercetagetin	45.01	0.31
MOL002757	*Carthami Flos*	7,8-Dimethyl-1H-pyrimido[5,6-g]quinoxaline-2,4-dione	45.75	0.19
MOL002773	*Carthami Flos*	Beta-carotene	37.18	0.58
MOL002776	*Carthami Flos*	Baicalin	40.12	0.75
MOL000006	*Carthami Flos*	Luteolin	36.16	0.25
MOL000098	*Carthami Flos*	Quercetin	46.43	0.28
MOL001771	*Carthami Flos*	Poriferast-5-en-3beta-ol	36.91	0.75
MOL000953	*Carthami Flos*	CLR	37.87	0.68
MOL001924	*Paeoniae Radix Alba*	Paeoniflorin	53.87	0.79
MOL000211	*Paeoniae Radix Alba*	Mairin	55.38	0.78
MOL000492	*Paeoniae Radix Alba*	(+)-Catechin	54.83	0.24
MOL001919	*Paeoniae Radix Alba*	(3S,5R,8R,9R,10S,14S)-3,17-Dihydroxy-4,4,8,10,14-pentamethyl-2,3,5,6,7,9-hexahydro-1H-cyclopenta[a]phenanthrene-15,16-dione	43.56	0.53
MOL000433	*Chuanxiong Rhizoma*	FA	68.96	0.71
MOL001494	*Chuanxiong Rhizoma*	Mandenol	42.00	0.19
MOL002135	*Chuanxiong Rhizoma*	Myricanone	40.60	0.51
MOL002140	*Chuanxiong Rhizoma*	Perlolyrine	65.95	0.27
MOL002157	*Chuanxiong Rhizoma*	Wallichilide	42.31	0.71

**Table 2:** Molecular docking results of hub targets and their related compounds.

Hub target	Compound	Combined energy (kcal/mol)
AKT1 (7NH5)	Kaempferol	-7.39
	Baicalein	-7.75
	Beta-carotene	-12.77
	Luteolin	-8.67
	Quercetin	-8.63

TP53 (5O1G)	Baicalein	-8.30

CASP3 (3DEF)	Beta-sitosterol	-7.19
	Kaempferol	-3.23
	Luteolin	-5.47
	Baicalein	-4.96
	Beta-carotene	-6.19
	Quercetin	-4.68

IL1B (5R86)	Quercetin	-7.49

JUN (4Y46)	Beta-sitosterol	-7.89
	Kaempferol	-5.07
	Luteolin	-7.31
	Beta-carotene	-8.99
	Quercetin	-6.44

## Data Availability

The data used to support the findings of this study are included within the article.
